# Precise Orchestration of Gasdermins' Pore-Forming Function by Posttranslational Modifications in Health and Disease

**DOI:** 10.7150/ijbs.86869

**Published:** 2023-09-18

**Authors:** Haiyang Jiang, Peizhao Liu, Jiaqi Kang, Jie Wu, Wenbin Gong, Xuanheng Li, Yangguang Li, Juanhan Liu, Weizhen Li, Chujun Ni, Bo Liao, Xiuwen Wu, Yun Zhao, Jianan Ren

**Affiliations:** 1 Department of General Surgery, Nanjing BenQ Medical Center, The Affiliated BenQ Hospital of Nanjing Medical University, Nanjing 210000, China.; 2 Research Institute of General Surgery, Affiliated Jinling Hospital, Medical School of Nanjing University, Nanjing 210000, China.; 3 Jiangsu Provincial Key Laboratory of Critical Care Medicine, Department of Critical Care Medicine, Affiliated Zhongda Hospital, School of Medicine, Southeast University, Nanjing, China.; 4 Department of General Surgery, The First Affiliated Hospital of Xi'an Jiaotong University, Xi'an 710061, Shaanxi Province, China.

**Keywords:** gasdermin, alternative splicing, pyroptosis, posttranslational modifications, dynamical regulation

## Abstract

Gasdermins (GSDMs) serve as pivotal executors of pyroptosis and play crucial roles in host defence, cytokine secretion, innate immunity, and cancer. However, excessive or inappropriate GSDMs activation is invariably accompanied by exaggerated inflammation and results in tissue damage. In contrast, deficient or impaired activation of GSDMs often fails to promptly eliminate pathogens, leading to the increasing severity of infections. The activity of GSDMs requires meticulous regulation. The dynamic modulation of GSDMs involves many aspects, including autoinhibitory structures, proteolytic cleavage, lipid binding and membrane translocation (oligomerization and pre-pore formation), oligomerization (pore formation) and pore removal for membrane repair. As the most comprehensive and efficient regulatory pathway, posttranslational modifications (PTMs) are widely implicated in the regulation of these aspects. In this comprehensive review, we delve into the complex mechanisms through which a variety of proteases cleave GSDMs to enhance or hinder their function. Moreover, we summarize the intricate regulatory mechanisms of PTMs that govern GSDMs-induced pyroptosis.

## Introduction

Two decades ago, gasdermins (GSDMs) were initially identified as risk genes associated with several alopecia-like skin diseases in mice and hearing loss in humans 1-4. The role of GSDMs in health and disease has been progressively elucidated since then. In humans, there are six GSDM genes: GSDMA, GSDMB, GSDMC, GSDMD, GSDME (also known as DFNA5), and PJVK (also known as DFNB59) 5, 6. Mice have three Gsdma genes (Gsdma1, Gsdma2, and Gsdma3), no Gsdmb gene, four Gsdmc genes (Gsdmc1, Gsdmc2, Gsdmc3, and Gsdmc4), and Gsdmd, Gsdme and Pjvk (Table 1). Previous research has established a strong correlation between GSDMs and sterile inflammation, as indicated by the association of Gsdma3 with alopecia induced by skin inflammation and that of GSDMB with childhood asthma 7-10. Further investigations have revealed that the deletion of exon 8 in GSDME is associated with multiple mutations that trigger programmed cell death in cochlear cells, indicating the potential cytotoxic activity of GSDMs 11-13. However, the specific type of cell death mediated by GSDMs and their underlying mechanisms remained unclear. Until 2015, GSDMD was considered executor of pyroptosis, which is a lytic form of cell death, and the substrate of proinflammatory caspases (caspase-1/-4/-5/-11) 14-16. The study of GSDMD activation and cleavage has led to the identification of activation mechanisms for other GSDMs, such as the caspase-3-GSDME and caspase-8-GSDMC pyroptotic axes 17, 18. Interestingly, several recent studies have revealed that cytotoxic lymphocytes interact with cells expressing GSDMB or GSDME to ultimately induce pyroptosis in these cells 19, 20. Exotoxin B (SpeB) is secreted by Group A Streptococcus and can cleave GSDMA and induce pyroptosis in keratinocytes 21, 22. These findings expand the scope of proteases activating GSDMs beyond intracellular caspases and further confirm that GSDMs serve as the executors of pyroptosis. The pore-forming and pyroptotic functions of GSDMs have gradually gained recognition.

**Figure 1:**
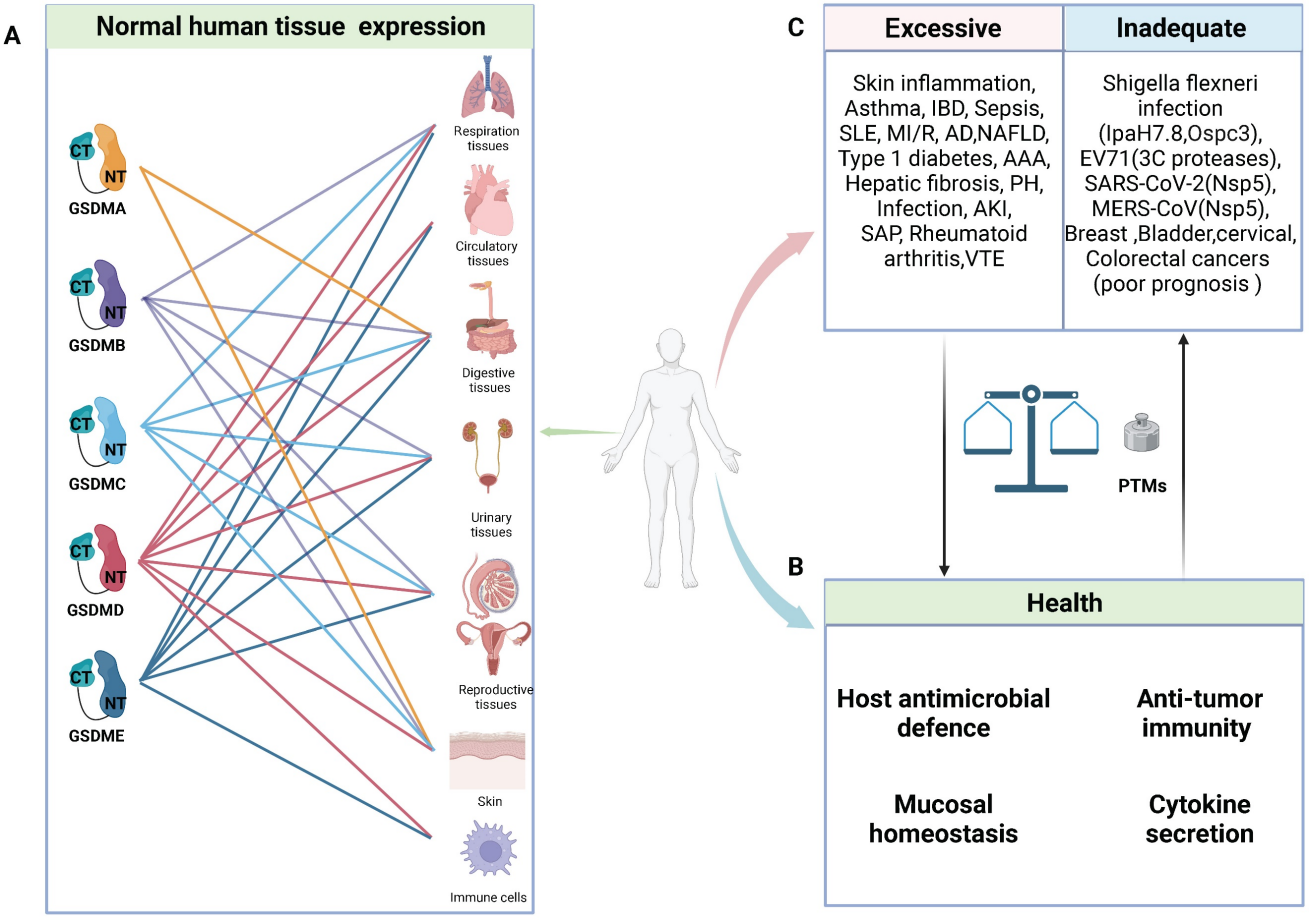
General characteristics of GSDMs in health and diseases. **A:** Schematic representation of normal expression of GSDMs in human tissues. **B:** In normal circumstance, GSDMs play a vital role in host anti-microbial defence, anti-tumor immunity, mucosal homeostasis and cytokine secretion. **C:** The pore-forming dysfunction of GSDMs is involved in the occurrence of various diseases. Abbreviations: IBD: inflammatory bowel disease; SLE: systemic lupus erythematosus; MI/R: myocardial ischemia/reperfusion; AD: Alzheimer's disease; NAFLD: non-alcoholic fatty liver disease; AAA: abdominal aortic aneurysm; PH: pulmonary hypertension; AKI: acute kidney injury; SAP: severe acute pancreatitis; VTE: venous thromboembolism; EV71: Enterovirus 71; PTMs: posttranslational modifications. Figures created with BioRender.com.

Due to their unique tissue expression and activation mechanisms, GSDMs can have multifaceted roles in health and disease (Figure 1). Numerous studies have demonstrated the pivotal roles of GSDMs in host defence. On the one hand, GSDMs can induce pyroptosis in infected cells to eliminate pathogen replication niches and subsequently recruit immune cells through the release of proinflammatory cytokines to combat pathogens 23, 24. On the other hand, GSDMB and GSDMD exhibit direct bactericidal activity *in vitro* by binding with bacterial membrane cardiolipin 25, 26. Furthermore, GSDMD and GSDME play crucial roles in mediating the release of IL-1β and NETosis, thereby promoting the elimination of *S. Typhimurium* and *Yersinia* in infected neutrophils 27-29.

Notably, certain invasive pathogens have developed intricate mechanisms to impair pyroptosis and sustain their replication, such as the truncation of pore-forming GSDMs and posttranslational modifications (PTMs) of GSDMs and their upstream caspases 25, 30-34. Nevertheless, every coin has two sides, and GSDM-induced pyroptosis is no exception. In animal experiments, pyroptosis inhibition or GSDMD knockout can effectively protect mice from fatal sepsis 35-37. This effect may be attributed to the excessive pyroptosis in epithelial cells leading to dysfunction in the intestinal barrier, ultimately resulting in worsened inflammation and injury. Furthermore, emerging evidence has unveiled a robust correlation between inflammatory ailments and pyroptosis. Various single-nucleotide polymorphisms (SNPs) in GSDMs have been shown to be linked with susceptibility to asthma, rheumatoid arthritis, and inflammatory bowel disease (IBD) (Table 1). The crucial roles of GSDMs as mediators of inflammation have been increasingly recognized, and targeting their function has become an attractive strategy for inhibiting inflammatory responses 38. However, cancer cell lysis and the intense inflammatory response mediated by GSDMs confer numerous benefits for antitumour therapy 39-42. In addition to directly inducing cancer cell lysis, the subsequent release of immunostimulatory cellular contents can effectively recruit and activate immune cells such as cytotoxic T cells, thereby facilitating tumour eradication 42. Thus, maintaining a delicate balance of pyroptosis is crucial in health and disease.

**Figure 2:**
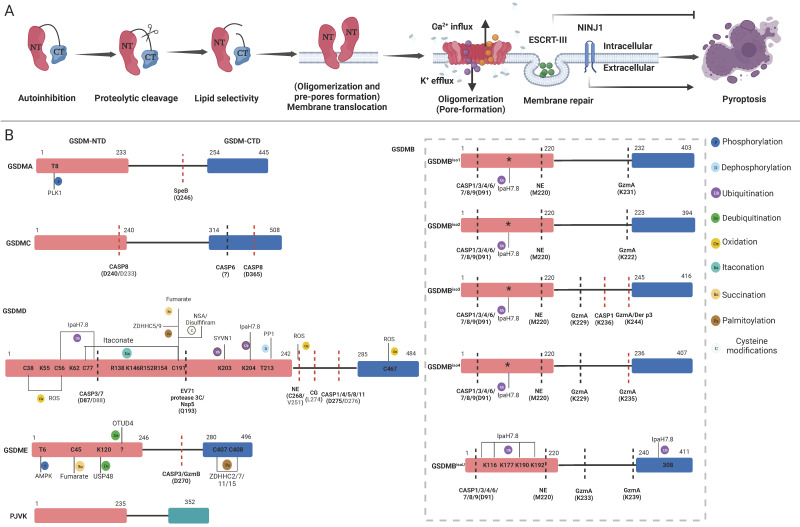
The pore-forming mechanism and regulatory sites of GSDMs. **A**: Main steps of GSDMs pore-forming. Autoinhibition: Interaction between the CTD and NTD of most GSDMs impedes protein activation. Proteolytic cleavage: Specific proteases cleave the link sites between the CTD and NTD of GSDMs, releasing pore-forming fragments. Lipid selectivity: Various GSDMs demonstrate distinct lipid-binding capacities and lipid preference. Membrane Translocation (Oligomerization and pre-pores forming): The GSDMs-NTD preassembling on the target membrane surface before membrane insertion. Oligomerizations (Pore-forming): GSDMs-NTD oligomerize and then form pore-like structures on membrane surface. Membrane repair: After pore-forming, ESCRT-III will be activated by cytosolic upregulated Ca2+ to excrete vesicles containing GSDMD pores to prevent further membrane rupture. The oligomerization of the membrane surface protein NINJ1 is vital for regulating the rupture of the membrane. **B**: Refining the pore-forming capabilities of GSDMs through cleavage and PTMs. Schematic representation of the structure of GSDMs present the proteases, respective cleavage sites, PTMs and PTMs sites. The sites in parentheses are proteases cleavage sites. The cleavage sites of murine proteins are indicated in grey letters within parentheses. Red lines mean the proteases can produce pore-forming NTD through cleaving these sites. Black lines mean the proteases inactivate GSDMs by cleaving these sites, releasing non-pyroptotic NTD. The PTMs sites are shown in the GSDMs-NTD and -CTD. Distinct modification types are distinguished by the use of various colors. The conserved ubiquitination sites of GSDMB isoforms are indicated by asterisks. Abbreviations: NTD: N-terminal domains; CTD: C-terminal domains; PTMs: posttranslational modifications; NINJ1: ninjurin 1; CASP: caspase; GzmA: granzyme A; GzmB: granzyme B; NE: neutrophil elastase; CG: cathepsin G; SYVN1: Synoviolin; NSA: necrosulfonamide; PP1: Phosphatase1; ROS: reactive oxygen species; ZDHHC: zinc finger and DHHC motif-containing family. Figures created with BioRender.com.

GSDMs-mediated pore formation is dynamically regulated at multiple levels through autoinhibitory structures, proteolytic cleavage, lipid binding and membrane translocation (oligomerization and pre-pore formation), oligomerization (pore formation) and pore removal for membrane repair 40, 43-51 (Figure 2A). Under normal circumstances, most GSDMs are located in the cytoplasm in an autoinhibited conformation 44. Except PJVK, all GSDMs contain two conserved domains: a C-terminal repressor domain (CTD) and an N-terminal domain (NTD). Specific proteases cleave linker sites, leading to the activation of GSDMs 15, 16. The GSDMs-NTD can specifically bind to lipids in the cell membrane, including phosphatidylinositol phosphates and phosphatidylserine (localized to the inner leaflet of the cell membrane), as well as cardiolipin (found in the inner and outer leaflets of bacterial membranes) 26, 48. Then, GSDMs-NTD oligomerizes and forms pre-pores and finally functional pores within membranes without the assistance of membrane receptors 52, 53. In contrast to mixed lineage kinase domain-like (MLKL)-mediated channel formation, which induces the influx of select ions to induce necroptosis, GSDMs-NTD oligomerizes and forms nonselective pores within membranes 54. Following GSDMs-mediated pores formation, cytosolic increases in Ca^2+^ activate the endosomal sorting complex required for protein transport (ESCRT)-III, which then releases vesicles containing GSDMD pores to prevent further membrane rupture and pyroptosis 46. In contrast, oligomers of the membrane surface protein ninjurin 1 (NINJ1), which contains two transmembrane domains, enhance plasma membrane rupture (PMR). In NINJ1-deficient cells, GSDMD pores are unable to induce cell rupture and typical pyroptosis 43, 47, 50. The strong lipid binding at the outer leaflet of the lipid bilayer indicates the underlying mechanism of NINJ1-mediated PMR. Although it forms ring-like structures, NINJ2, a paralogue of NINJ1, failed to mediate PMR due to its binding to cholesterol in the inner leaflet 55. Currently, the model of NINJ1-mediated PMR remains controversial 43, 47, 55, 56. The release of NINJ1 oligomers into the supernatant challenges the amphipathic filament model or pore model with a hydrophilic conduit, which may support the cookie cutter model of cell lysis 55, 56. Understanding whether NINJ1 forms rings with hydrophobic interiors and hydrophilic exteriors or pores containing a hydrophilic conduit is worth further investigation. In summary, these dynamic processes present promising targets for modulating pyroptosis.

PTMs, which include but are not limited to phosphorylation, dephosphorylation, ubiquitination, deubiquitination, oxidation, itaconation, succination, and palmitoylation, exert profound effects on various aspects of GSDM-related protein function by precisely regulating specific amino acids within proteins. To date, various strategies for regulating GSDM signalling through PTMs have been elucidated, including GSDMs activation, proteolytic cleavage, membrane translocation, GSDMs-NTD oligomerization and other nonpyroptotic functions. This review will provide a comprehensive discussion on the fundamental biological mechanisms of GSDMs-mediated pyroptosis, recent advances in PTMs of GSDMs, and potential therapeutic applications based on PTMs for infectious diseases, cancer, and other inflammatory disorders.

## Gasdermins' Characteristics and Cleavage Mechanisms

### GSDMA

GSDMA is predominantly expressed in keratinocytes in the skin and epithelial cells in the gastrointestinal tract and has been linked to various dermatological conditions, systemic sclerosis, asthma, and IBD (Table 1). Previous research has indicated that GSDMA is linked to mitochondrial stress and dysfunction 57. Consistently, a protein engineering study demonstrated that upon activation, GSDMA localized to mitochondria and showed delayed and decreased accumulation at the plasma membrane 58. GSDMA-NTD induces early mitochondrial dysfunction prior to plasma membrane disruption, suggesting interplay between pyroptosis and cell death that centres on mitochondria.

Recent studies have uncovered a novel role of GSDMA in host recognition and the maintenance of defence barriers 21, 22. Cleavage of the linker site of GSDMA at Q246 by SpeB leads to pyroptosis in keratinocytes (Figure 2B). Deng et al. and LaRock et al. used GAS to infect Gsdma1-knockout and Gsdma1-3-triple-knockout mice and confirmed that GSDMA plays a pivotal role in preventing colonization and infection by GAS. Following the transfection of GSDMs into HEK293T cells and incubation with SpeB, immunoblot analysis revealed signs of cleavage in GSDMC and GSDMD. In contrast, Deng et al. reported that coexpression of SpeB and GSDMA-E in 293T cells resulted in the specific cleavage of GSDMA but not GSDMB-E. It is worth exploring the potential of SpeB to cleave GSDMC and GSDMD, as well as elucidate the circumstances under which it exerts its proteolytic effects. Under normal conditions, autophagic machinery can effectively eliminate intracellular GAS through lysosomal degradation 59. However, by secreting SpeB, GAS evades this innate immune defence mechanism. As an alternative strategy, SpeB-activated GSDMA induces lysis in infected host cells to restrict pathogen replication. Given their intracellular localization, GSDMs likely serve as sentinels against other internalized microbes.

### GSDMB

GSDMB is predominantly expressed in the gastrointestinal tract, airway epithelium, lymphoid tissues, and various tumours^60^, ^61^. Genome-wide association studies have demonstrated a close correlation between GSDMB polymorphisms and susceptibility to asthma, IBD, and other chronic inflammatory diseases (Table 1).

Over the years, the functions and activation pathways of GSDMB have been a subject of dispute due to its unique lipid-binding properties and complex isoforms that result in differing crystal structures 62-64. GSDMB is encoded by various splice variants, each with their own counterparts (Table 1). The isoforms of GSDMB exhibit variations in the presence or absence of exons 6 and 7. Specifically, GSDMB^iso1^ and GSDMB^iso4^ lack exons 6 and 7, respectively; GSDMB^iso2^ lacks both exons, while GSDMB^iso3^ contains both exons 65. In addition to GSDMB^iso1-4^, several studies have used GSDMB^isoU^ to investigate the nonpyroptotic functions of GSDMB. GSDMB^isoU^ is similar to GSDMB^iso4^ but has an asparagine-to-aspartate substitution in the first residue in exon 6, in front of which is a four-residue insert 25, 65, 66. Until recently, two pioneering studies used cryogenic electron microscopy to determine the indispensable role of exon 6 amino acids in the linker sequences of GSDMB isoforms in pore assembly and phospholipid binding 65, 67. Several contemporaneous studies have also demonstrated that the presence of exon 6 is pivotal for defining the activity of GSDMB isoforms 68, 69. Moreover, Kong et al. discovered that the coexpression of noncytotoxic isoforms (GSDMB^iso1/2^) could inhibit the pore-forming activity of cytotoxic isoforms (GSDMB^iso3/4^) 68. This phenomenon may be attributed to the formation of hetero-oligomers, which inhibit crucial steps from intermediate assemblies to membrane pore formation (Figure 4). The composition of GSDMB isoforms varies in distinct cells and contexts and modulates the final pore-forming activity. Therefore, distinguishing between the pore-forming activity and tissue expression of different isoforms is crucial for elucidating the function of GSDMB.

Although GSDMB^iso1^ and GSDMB^iso2^ possess several protease cleavage sites, their ability to induce pyroptosis through pore formation is limited due to the absence of exon 6 (Table 1). Several earlier studies reported the noncytotoxic function of GSDMB^iso1^. Panganiban et al. found that the coding variant rs11078928 regulated the exon-5-8 transcript of the GSDMB gene and failed to induce pyroptosis via caspase-1 because exon 6 was skipped, which was associated with a decrease in asthma risk 70. Consistently, a recent study reported that Der p3 from house dust mites (HDMs) can directly cleave GSDMB^iso3^ to induce human bronchial epithelial (HBE) cell pyroptosis. However, GSDMB^iso1^-NTD failed to mediate HBE cell pyroptosis 71. In a separate study on asthma, GSDMB^iso1^ was identified as the predominant isoform and a transcriptional activator of TGFβ, MMP9, and chemokines in bronchial epithelial cells 72. This finding suggests that GSDMB acts as a transcription activator rather than an executor of pyroptosis. Das et al. created a knock-in mouse model of human GSDMB^iso1^. After methacholine exposure, GSDMB^iso1^ knock-in mice exhibited heightened airway responsiveness and more severe airway remodelling. However, compared to wild-type mice, there was no change in inflammatory cells in bronchoalveolar lavage fluid 72. These findings suggest that different isoforms of GSDMB may be involved in asthma through distinct mechanisms. Subsequent studies have provided compelling evidence that the cytoplasm and nuclei of HeLa and HEK293T cells contain GSDMB^iso1^ and its NTD. In contrast to its role in bronchial epithelial cells, GSDMB^iso1-4^ did not function as a transcription factor in HeLa cells 68. These findings suggest that the nonpyroptotic role of GSDMB as a transcription factor is dependent on specific isoforms and conditions.

Whether caspases are capable of cleaving GSDMB^iso3^ and GSDMB^iso4^ to induce pyroptosis remains a controversial topic. Panganiban et al. demonstrated that caspase-1 could cleave GSDMB^iso3^ at D236, which is located in exon 7, leading to the induction of pyroptosis in HEK293T cells 70. Conversely, several studies showed that caspase-1/3/4/6/7/8/9 and NE mediated proteolytic inactivation of GSDMB^iso3^ and GSDMB^iso4^ in THP-1 cells 63, 69, 73 (Figure 2B). Moreover, full-length GSDMB^iso3^ was shown to promote GSDMD-mediated noncanonical pyroptosis by strengthening the activity of caspase-4 in THP-1 macrophages 73. The intricate interplay between GSDMB and caspases poses a challenge to determining the pyroptotic functions of GSDMB^iso3^ and GSDMB^iso4^. In 2020, Zhou and colleagues reported that granzyme A (GzmA) derived from natural killer (NK) and/or CD8^+^ T cells cleaved GSDMB at K244 (major cleavage site) and K229 (minor cleavage site), which was conserved in GSDMB^iso1-4^ in a colon cancer cell line, resulting in pyroptosis in cancer cells 20. A subsequent study showed that only GzmA cleaved GSDMB^iso3^ at K244 and that GSDMB^iso4^ could exhibit pore-forming activity. Compared to that of GSDMB^iso3^, the cytotoxicity of GSDMB^iso4^ was relatively weak 68. Analogously, another study showed that GSDMB^iso4^ and GSDMB^iso3^ derivative fragments that lacked exon 7 exhibited only partial pore-forming activity 65. After being attacked by NK cells, GSDMB^iso1-2^-expressing cells tended to undergo apoptosis, while GSDMB^iso3^-expressing cells predominantly underwent pyroptosis. In contrast, GSDMB^iso4^-expressing cells underwent mixed pyroptosis and apoptosis within tumours 68. Increasing the expression of GSDMB^iso3^ and/or GSDMB^iso4^ is a crucial strategy for antitumour therapy due to the immunogenic inertness of apoptosis compared to pyroptosis. Indeed, recent studies have reported distinct functions of GSDMB^iso1-4^ in various cancers. Notably, the high expression of pyroptotic GSDMB^iso3^ or GSDMB^iso4^ but not GSDMB^iso1^ or GSDMB^iso2^ has been associated with a more favourable prognosis among patients with bladder, breast and cervical cancers 68, 69. Furthermore, GSDMB^iso3^ and GSDMB^iso4^ account for 75% of total GSDMB transcripts in small intestinal mucosal and rectal epithelial cells, as well as over 38% of total GSDMB transcripts in colonic epithelial cells, indicating that GSDMB-mediated pyroptosis may play a pivotal role in gastrointestinal diseases such as IBD 65.

Two recent studies reported the nonpyroptotic functions of GSDMB using a GSDMB isoform recorded in the UniProt database (termed GSDMB^isoU^) 25, 66. Hansen et al. discovered that GSDMB^isoU^ was cleaved by GzmA and preferred to form pores in bacterial-derived membranes by constructing bacterial-mimetic liposomes and mammalian liposomes *in vitro*. The authors reached the same conclusion in GSDMB^isoU^ knock-in mice that GSDMB^isoU^ was cleaved by GzmA and directly killed intracellular bacteria instead of inducing host cell pyroptosis 25. However, GSDMB^isoU^ has negligible pore-forming activity compared with GSDMB^iso3^ due to a four-residue insertion before exon 6 that disarranges the β10 structure of GSDMB^isoU^-NTD 65. This finding suggests that GSDMB may exert significant effects on host antimicrobial defence, although the pore-forming activity of GSDMB isoforms differs. Subsequent studies showed that different NTDs of GSDMB isoforms similarly bound to cardiolipin and other lipids. However, only GSDMB^iso3^-NTD244 (cleaved at K224) and GSDMB^iso4^-NTD killed bacteria *in vitro* 68. Currently, investigations of the bactericidal functions of GSDMB isoforms are primarily based on *in vitro* studies, and further animal experiments are required to improve our understanding. Moreover, GSDMB^isoU^ affects epithelial maintenance and repair by promoting the proliferation, migration, and adhesion of intestinal epithelial cells (IECs) in full-length (FL) form, which regulates PDGF-A-dependent FAK phosphorylation 66. However, the expression of GSDMB^isoU^ is minimal in normal IECs 65. Whether and how isoform composition changes in IBD remain unknown, indicating that the nonpyroptotic function of GSDMB in IBD requires further study. In addition, the association between changes in the GSDMB isoform and different disease phenotypes of IBD, such as active intestinal inflammation and fibrosis, is also worth exploring.

The isoforms collectively play a crucial role in the biological functions of GSDMB in various contexts. It is imperative to determine which GSDMB isoform is predominant in specific cells and how isoform composition changes in health and disease conditions.

### GSDMC

GSDMC is widely expressed in various tissues, including the upper gastrointestinal and airway epithelium, skin, and spleen 60. In addition to inducing pyroptosis, GSDMC has been shown to participate in the type 2 immune response to helminth infections by promoting unconventional secretion of IL-33 through GSDMC pores 61, 74, 75 (Table 1 and Figure 4).

Recently, Hou et al. reported that caspase-8 cleaves GSDMC at D365 in breast cancer cells, leading to a switch from TNFα-induced apoptosis to pyroptosis 17 (Figure 1B). Under hypoxic conditions, STAT3 and PD-L1 translocate to the nucleus to upregulate GSDMC expression. Ultimately, caspase-8 cleaves GSDMC to generate GSDMC-NTD, inducing pyroptosis in breast cancer cells. In addition to that of caspase-8, the ability of caspase-6 to cleave GSDMC has also been discovered. Interestingly, GSDMC cleaved by caspase-6 failed to induce pyroptosis in response to treatment with TNFα plus CHX. It would be worthwhile to investigate whether caspase-6 can induce pyroptosis by cleaving GSDMC under conditions of caspase-6 activation 17. Additionally, the metabolite α-ketoglutarate (α-KG) has been reported to increase intracellular ROS levels and activate the plasma membrane-localized death receptor DR6, which serves as a protein‒protein interaction platform for caspase-8 and GSDMC 76 (Figure 4). Under different conditions, including different cell lines and stimuli, caspase-8 may cleave GSDMC at alternative sites (D240) to release the pyroptotic NTD 76. However, the underlying regulatory mechanism requires further investigation.

### GSDMD

GSDMD is widely expressed in various tissues and is involved in multiple inflammatory diseases and cancers 41, 61, 77-79. GSDMD pores not only induce lytic cell death but also serve as channels for the release of inflammatory cytokines 80-82. Additionally, GSDMD plays a crucial role in maintaining intestinal mucosal homeostasis by promoting mucus layer formation to defend against various pathogens 83 (Table 1).

**Figure 3:**
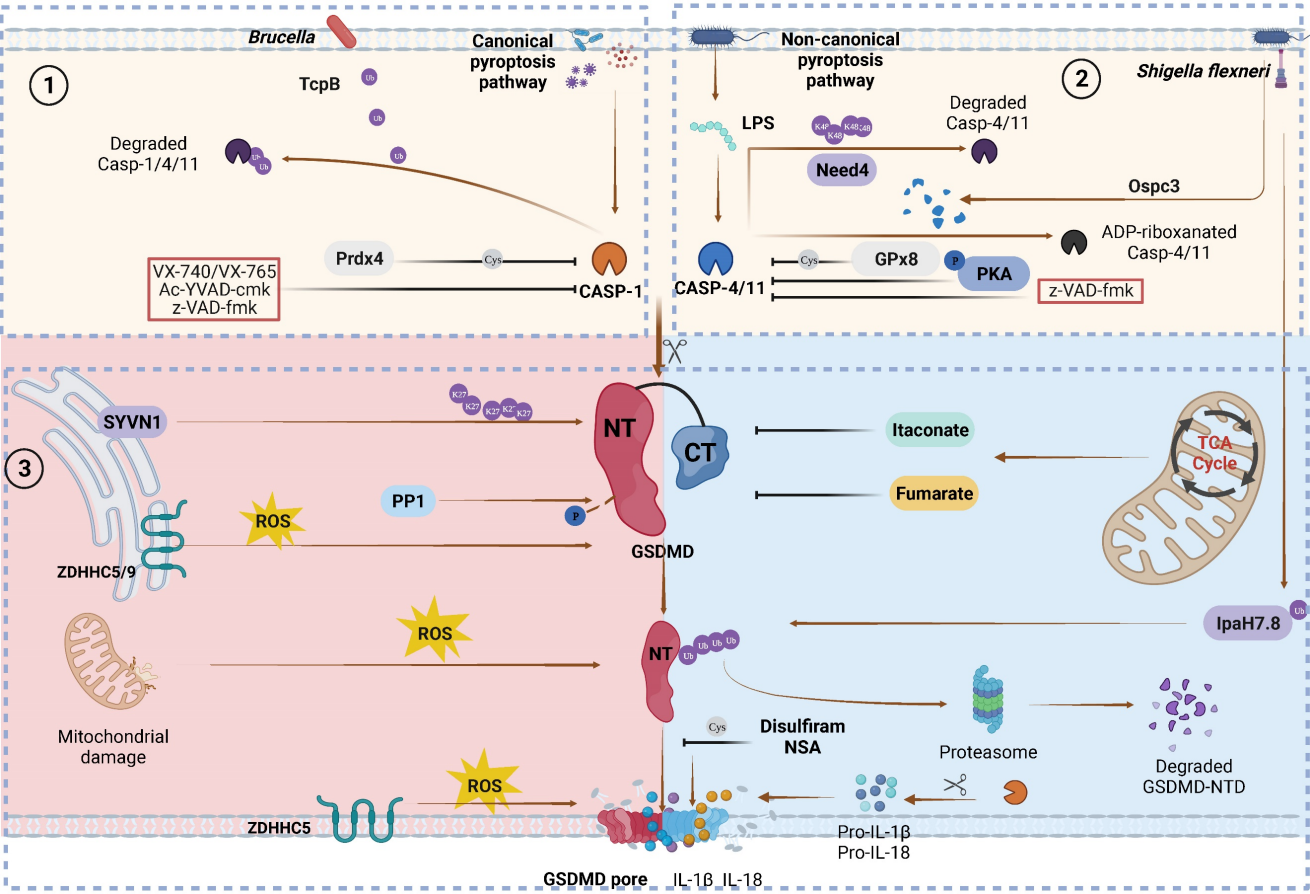
Canonical and non-canonical GSDMD cleavage and PTMs regulation. Caspase PTMs regulation. (1) TcpB secreted by* Brucella* to ubiquitylates caspase-1/4/11 and promotes their degradation, leading to inactivate GSDMD signaling. Prdx4 inactivates caspase-1 by modifying its C397. VX740, VX765, Ac-YVAD-cmk and z-VAD-fmk covalently modify the catalytic cysteine residues to inactivate caspases. (2) Nedd4 induces the K48-linked polyubiquitination of caspase-11 and succeeding degradation. PKA phosphorylates caspase-11, inhibiting non-canonical pyroptosis. GPx8 negatively regulated activation of GSDMD via disulfide bounding to the C118 site in caspase-4/11. *Shigella flexneri* can secrete OspC3 to inhibit pyroptosis via the ADP-ribosylation modification of caspase-4 and caspase-1l respectively at R314 and R310. GSDMD PTMs regulation. (3) SYVN1 induces K27-linked polyubiquitination of GSDMD at K203 and K204, promoting pyroptosis. PP1 enhances pyroptosis by dephosphorylating multiple sites on GSDMD. ZDHHC5/9 facilitates GSDMD ROS-dependent palmitoylation at C191 human (C192 mouse), promoting plasma membrane localization. ROS promote macrophage pyroptosis by oxidizing GSDMD. Endogenous itaconate covalently binds to GSDMD at C77, reducing caspase-1-mediated GSDMD cleavage. Fumarate can bind to GSDMD at multiple sites called succination to reduce proteolytic cleavage and inhibit NTD oligomerization. Disulfiram /NSA can inhibit NTD oligomerization through cys-modifying. *Shigella flexneri* can secrete IpaH7.8 to ubiquitylate hGSDMD and promote it degradation to inhibit pryroptosis. Abbreviations: Prdx4: thiol-specific peroxidase peroxiredoxin-4; PKA: Protein kinase A; GPx8: Glutathione peroxidase 8. Figures created with BioRender.com.

In canonical inflammasome pathways, cytosolic pattern recognition receptors (PRRs) specifically monitor pathogen-associated molecular patterns (PAMPs) and damage-associated molecular patterns (DAMPs) from intracellular and extracellular sources (Figure 3). Subsequently, these receptors directly recruit downstream pro-caspase-1 or apoptosis-associated speck-like protein containing a caspase recruitment domain (ASC) to further recruit pro-caspase-1, resulting in the formation of inflammasome complexes 84. Inflammasome complexes activate caspase-1, which in turn promotes the maturation of proinflammatory precursor cytokines (pro-IL-1β and pro-IL-18) and proteolytic cleavage of GSDMD 85. In the noncanonical inflammasome pathway, caspase-11 in mice (caspase-4 and caspase-5 in humans) can be activated by direct recognition of intracellular lipopolysaccharide (LPS) from gram-negative bacteria 86.

**Figure 4:**
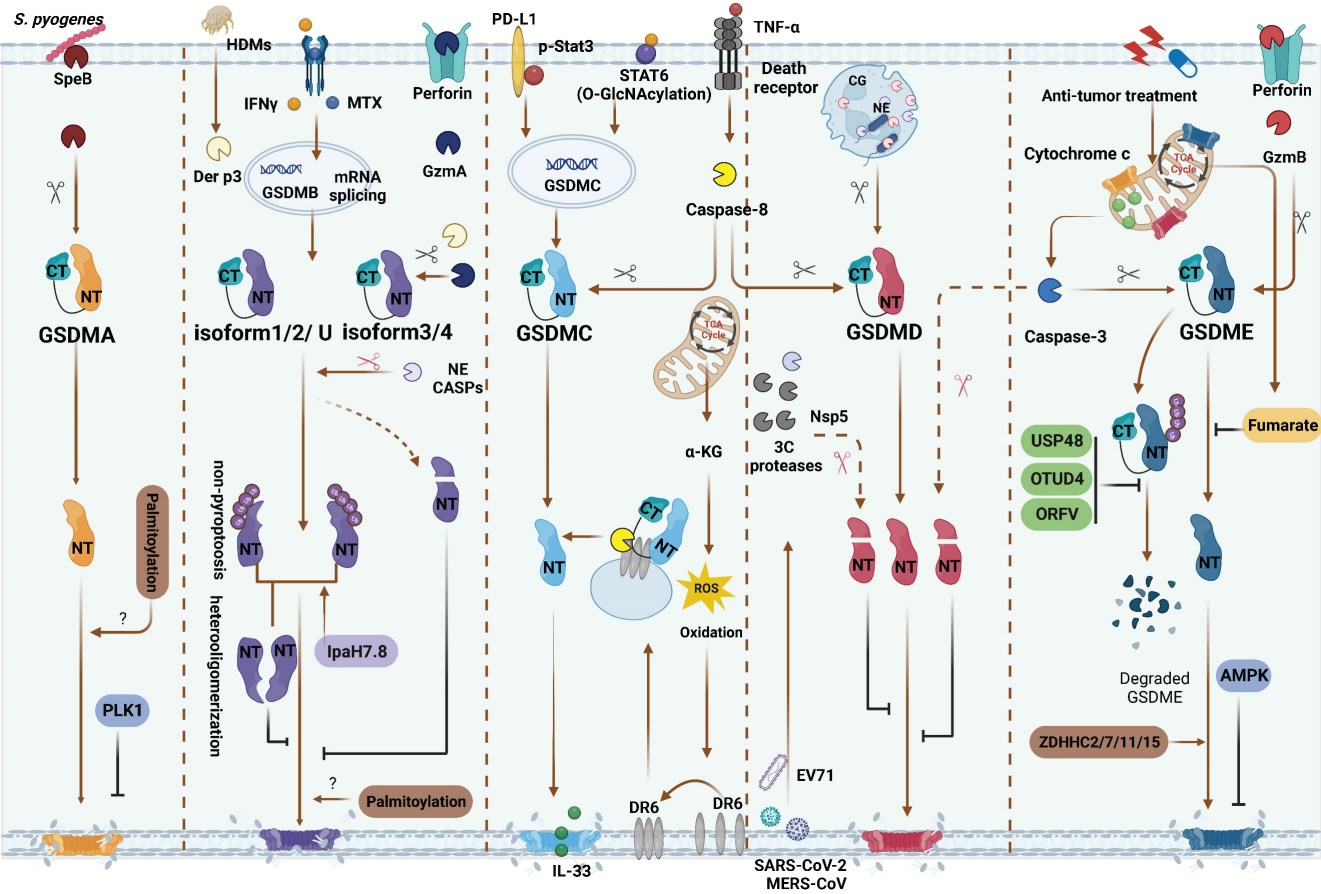
Inflammasome-independent GSDMs cleavage and PTMs regulation. GSDMA: SpeB directly activate GSDMA. PLK1 mediate GSDMA phosphorylation and inhibit GSDMA-NTD oligomerization. GSDMB: Methotrexate and IFNγ are identified as inflammatory or non-inflammatory stimuli of upregulating various GSDMB isoforms, respectively. GzmA from NK and/or CD8^+^ T cells and Der p3 from HDM can cleave different GSDMB isoforms. Only GzmA cleave GSDMB^iso3,4^ and Der p3 cleave GSDMB^iso3^ at K244 can produce pyroptotic NTD. Caspase-1 also can cleave GSDMB^iso3^ at D236 to induce pyroptosis. Caspase-1/3/4/6/7/8/9 and NE mediated proteolytic inactivation of GSDMB^iso1-4^. Non-pyroptotic GSDMB-NTD can inhibit the pore-forming activity of pyroptotic GSDMB-NTD by heterooligomerization. *Shigella flexneri* can secrete IpaH7.8 to ubiquitylate almost GSDMB isoforms and induce them degradation or directly inhibit the pyroptosis functions of GSDMB. GSDMC: In hypoxia situation, STAT3 and PD-L1 migrated to nucleus, enhancing GSDMC expression. TNFα-induced caspase-8 cleaves GSDMC in cancer cells. In response to helminth infection, STAT6 O-GlcNAcylation induces GSDMC expression. Then caspase-8 activates GSDMC and forming the pores for IL-33 releasing. α-KG can elevate intercellular ROS level to activate plasma membrane-localized death receptor DR6 serving as a platform caspase-8 and GSDMC interaction. GSDMD: In neutrophils, CG and NE activate GSDMD to induce NETosis. 3C protease from EV71, Nsp5 from SARS-CoV-2, MERS-CoV and caspase-3 can inactivate GSDMD. GSDME: Caspase-3 and GzmB can cleave GSDME at the same site. AMPK phosphorylates GSDME at T6 and inhibits pyroptosis. Fumarate can bind to GSDME at multiple sites called succination to inhibit pyroptosis. In anti-tumor therapies, USP48, OTUD4 and ORFV can reduce GSDME ubiquitination and degradation. During chemotherapy, ZDHHC2/7/11/15 can promote GSDME palmitoylation and further pyroptrosis. Red scissors represent inactivation and black scissors represent activation. Abbreviations: SpeB: *Streptococcus pyogenes* exotoxin B; PLK1: Polo-like kinase 1; CASP: caspase; HDMs: house dust mites; NE: neutrophil elastase; CG: cathepsin G; α-KG: α-ketoglutarate; DR6: death receptor 6; EV71: Enterovirus 71; AMPK: AMP-activated protein kinase; OTUD4: ovarian tumor family deubiquitinase 4; ORFV: oncolytic parapoxvirus ovis. Figures created with BioRender.com.

In addition to proinflammatory caspases (caspase-1/-4/-5/-11), other caspases can process GSDMD into active or inactive fragments (Figure 4). Recent studies have revealed that the *Yersinia* effector protein YopJ can inhibit TGFβ-associated kinase 1 (TAK1) to promote apoptotic caspase-8 cleavage of GSDMD at D276 (human D275) in murine macrophages, ultimately leading to pyroptosis^87^, ^88^. This finding suggests that the caspase-8-GSDMD pathway serves as an emergency host defence mechanism that induces pyroptosis in the absence of proinflammatory caspases. Furthermore, in the event of gram-negative bacterial infection of neutrophils, the granule-associated proteases cathepsin G (CatG) and ELANE (neutrophil elastase), which are specific to neutrophils, can also process GSDMD into active GSDMD-NTD (Figure 2B), thereby inducing pyroptosis in neutrophils and leading to the formation of neutrophil extracellular traps (NETs) as a means of preventing further infection 89, 90.

In addition to cleaving GSDMD to induce pyroptosis, certain caspases or proteases from pathogens can mediate the proteolytic inactivation of GSDMD. Apoptotic caspase-3 and caspase-7 can cleave D87 (mouse D88), which is located within the pore-forming GSDMD-NTD, resulting in a shortened GSDMD-NTD that has lost its ability to induce pyroptosis 91-93 (Figure 2B). In addition to host-derived proteases, GSDMD is also inactivated by exogenous proteases produced by pathogens to inhibit pyroptosis. 3C proteases, which are viral proteins produced by enterovirus 71 (EV71), process GSDMD at Q193 to inhibit pyroptosis, suggesting a strategy by which invading pathogens evade the host immune system 31. Likewise, the 3C-like protease Nsp5 produced by coronaviruses (CoVs) such as SARS-CoV-2, MERS-CoV, PDCoV, and PEDV can cleave human GSDMD (hGSDMD) at Q193 to generate two nonpyroptotic fragments. This results in an inability to impair viral replication 94 (Figure 4).

Activated GSDMD can also bind cardiolipin, which is primarily expressed in the inner and outer leaflets of bacterial membranes, to directly kill bacteria 26. Similarly, GSDMD-NTD regulates mitochondrial function by binding to cardiolipin and forming pores in the mitochondrial membrane structure, resulting in mitochondrial oxidative stress and the release of mitochondrial dsDNA (mtDNA) 53, 57, 95 (Figure 4). Recently, the identification of GSDMD pore formation in mitochondria and the release of mtDNA have been recognized as prerequisites for Nur77-mediated detection of intracellular LPS and dsDNA, thereby activating the noncanonical NLRP3 inflammasome 96. This finding highlights the extensive interactions between GSDMD and inflammation, as well as various cell death types, including pyroptosis, apoptosis, and necroptosis. Regulating pore formation by GSDMD is crucial for maintaining immune homeostasis and treating inflammatory diseases.

### GSDME

GSDME is extensively expressed in various tissues, including the cochlea, skin, and gut. Initially, identified as a contributor to human hearing loss, GSDME has since been linked to inflammatory diseases such as atherosclerosis, skin inflammation, and IBD 60, 61. Additionally, its unique ability to switch from apoptosis to pyroptosis holds great promise for tumour immunotherapy (Table 1).

Caspase-3 can cleave GSDME at D270, thereby generating the pyroptotic GSDME-NTD and inducing cell death. In response to chemotherapy drugs or apoptotic triggers, cells expressing GSDME undergo a transition from apoptosis to pyroptosis 18, 97. Zhang et al. revealed that granzyme B (GzmB), which is s serine protease secreted by cytotoxic T lymphocytes and NK cells, triggers GSDME-dependent pyroptosis in the tumour microenvironment by cleaving GSDME at the same site as caspase-3 19 (Figure 2B). Recent studies have revealed the pivotal role of GSDME in IBD 98, 99. Increased levels of GSDME are cleaved by caspase-3, leading to IEC pyroptosis and the subsequent release of proinflammatory cytokines that augment immune responses 99. Moreover, in a mouse model that was intravenously injected with TNFα, GSDME and upstream interferon regulating factor 1 (IRF1) were crucial for IEC shedding in IBD 98. Moreover, the importance of GSDME in defending against viral and *Candida albicans* infections has been demonstrated 100-102. In T cells, keratinocytes and alveolar epithelial cells, pathogen invasion results in mitochondrial damage, which subsequently triggers caspase-3 cleavage of GSDME and culminates in IL-1α release and cell death. Mitochondrial damage is a ubiquitous process that eukaryotes undergo in response to endogenous or exogenous stimuli, suggesting that the caspase-3-GSDME axis may be a general mechanism during abnormal conditions 103, 104. Furthermore, GSDMA and GSDMD can target the mitochondrial membrane, and GSDMB induces pyroptosis with mitochondrial damage 53, 57, 58, 69, 95. Whether these GSDMs interact with GSDME remains unclear, and more studies are needed to further explore the possible relationships.

### PTMs: The regulators of GSDMs functions

The precise regulation of GSDMs-mediated pore formation plays a pivotal role in determining cell fate. As the most ubiquitous and efficient regulatory pathway, PTMs extensively participate in the modulation of protein stability, localization, and protein‒protein interactions by covalently attaching specific chemical groups to amino acid side chains of target proteins 105-107. PTMs exert a range of regulatory effects on pyroptosis. In the subsequent sections, we will examine the distinct PTMs of each GSDM and elucidate their specific roles in various pathophysiological contexts.

### Phosphorylation and dephosphorylation

Phosphorylation and dephosphorylation are common mechanisms for regulating protein activity 108. GSDMA and GSDME undergo phosphorylation at T8 and T6, respectively, by unidentified kinases (Table 2). Phosphorylation of the GSDMA and GSDME NTDs inhibits the oligomerization of GSDMs and pyroptotic activity 109. A PLK1-dependent phosphoproteome was identified, indicating that Polo-like kinase 1 (PLK1), a serine-threonine kinase, may be involved in the phosphorylation of GSDMA 110 (Figure 4). However, it remains unclear whether PLK1 phosphorylates GSDME in a similar manner. In addition, some metabolites with kinase activity may exert broad functions and regulate pyroptosis. AMP-activated protein kinase (AMPK) is activated by the metabolite N-acetylglucosamine-6-phosphate (GlcNAc-6P) and antagonizes GSDME-mediated pyroptosis by phosphorylating GSDME at T6 111. Recently, Li et al. reported that the phosphorylation of GSDMD at T213 could regulate oligomerization and suppress pyroptosis through steric hindrance (inhibiting the interaction between GSDMD monomers), while phosphatase 1 (PP1) can dephosphorylate multiple sites on GSDMD including T213 to increase pyroptosis 112. Based on the phosphoproteome and proteomic mass spectrometry, GSDMD has several potential phosphorylation sites whose impact on its function remains unclear (Table 2). The pore-forming activity of GSDMs may be limited by phosphorylation, which is dependent on the structural characteristics of GSDMs.

### Ubiquitination and deubiquitination

Protein ubiquitination is a dynamic PTM involving the covalent attachment of ubiquitin to a target protein and includes monoubiquitination and polyubiquitination 113. The effects of this modification vary depending on the type of ubiquitination and substrate involved.

Stabilizing or inhibiting GSDM degradation can enhance the effects of pyroptosis. Shi et al. found that Synoviolin (SYVN1) promotes pyroptosis by inducing K27-linked polyubiquitination of GSDMD at K203 and K204 114 (Figure 3). Ubibrowser software was used to investigate other potential E3 ubiquitin ligases that interact with GSDMD, such as Mindbomb Homologue 2 (MIB2) and Nedd4. Another study showed that sodium arsenite (NaAsO2) prevented GSDMD degradation by inhibiting K48- and K63-linked ubiquitination, leading to GSDMD accumulation and pyroptosis 115. Consequently, uncontrolled pyroptosis worsens liver damage and insulin resistance. Moreover, some E3 ligase proteins can interact with GSDMs without using their ligase activity. For example, the PRY-SPRY domain of TRIM21 binds to GSDMD to stabilize and promote NTD oligomerization independently of its E3 ligase activity 116.

In cancer treatments, the deubiquitination of GSDMs through activation and stabilization is a valuable strategy (Figure 4). Ovarian tumour family deubiquitinase 4 (OTUD4) can enhance radiosensitivity in nasopharyngeal carcinoma cells by positively regulating pyroptosis via GSDME deubiquitination 117. Similarly, USP48 can deubiquitinate GSDME at K120 and K189 by removing the K48-linked ubiquitin chains, thereby augmenting cancer cell susceptibility to pyroptosis in response to treatment 118. Another investigation demonstrated that oncolytic parapoxvirus ovis (ORFV), a promising biotherapeutic antitumour agent, promotes tumour cell pyroptosis by decreasing GSDME ubiquitination 119. While some deubiquitinating enzymes can stabilize and promote GSDME to induce lytic cell death, others may have the opposite effect on cancer cells. For example, in bladder cancer, USP24 stabilizes the nonpyroptotic GSDMB isoform, which enhances STAT3 phosphorylation and promotes cancer cell proliferation 120.

In response to pathogenic bacteria invading the host, GSDMs quickly execute pyroptosis in infected cells such as epithelial cells, neutrophils, and macrophages to prevent intracellular pathogen replication 23, 28, 121-123. However, certain microorganisms have developed specific mechanisms to counteract pyroptosis 124, 125 (Figure 3,4). Hansen et al. discovered that IpaH7.8, an E3 ubiquitin ligase produced by *Shigella flexneri*, targets K166 and K308, as well as other ubiquitinated residues of GSDMB^isoU^, to induce 26S proteasomal degradation, thereby protecting *Shigella flexneri* from the bactericidal activity of NK cells 25. A subsequent study showed that ubiquitination of GSDMB at K177, K190 and K192 by IpaH7.8 was sufficient to block GSDMB-induced pyroptosis without the involvement of the proteasome 67. Interestingly, several studies demonstrated that IpaH7.8 could inhibit the pyroptotic activity of hGSDMD but not mouse GSDMD (mGSDMD) through the ubiquitination of K55, K62, K204 and other residues 33, 126. The presence of R20 in mGSDMD creates a bulge in its crystal structure, which prevents IpaH7.8-mediated ubiquitination compared to hGSDMD 126. Moreover, several studies have reported that pathogens can disrupt host defence by inducing ubiquitination, ADP-ribosylation, and ADPR-deacylization in GSDM-mediated pyroptosis pathway factors, suggesting that PTMs may be exploited by invading pathogens 30, 32, 34 (Figure 3).

### Oxidation

Oxidation‒reduction reactions play a crucial role in cellular metabolism, and their by-products regulate various biological processes, such as stress responses, inflammation, and cell death, through multiple pathways 127. Exogenous and endogenous ROS can activate inflammasome pathways and stimulate pyroptosis 128, 129. During pore formation, the GSDMD-NTD cannot induce pyroptosis without the Ragulator-Rag components RagA or RagC. However, this can be remedied with ROS 130. Moreover, ROS can exert their effects through the covalent modification of proteins. Recent studies have revealed that oxidative PTMs involving cysteines within GSDMD are closely associated with pyroptosis (Figure 3). In 2019, Wang et al. discovered that mitochondrial ROS (mtROS) induced macrophage pyroptosis by oxidizing hGSDMD at C38, C56, and C268 and mGSDMD at C39, C57, C265 and C487 131. Another recent study reported that during pyroptosis, the C57, C77, C192 and C265 sites of mGSDMD-NTD underwent oxidation 132 (Table 2). Among these sites, C192 was indispensable for ROS responsiveness.

In addition to activating inflammasome pathways and inducing oligomerization through oxidative modification of GSDMD-NTD, mtROS may possess other mechanisms by which they regulate pyroptosis. (Table 2). A recent study showed that oxidized mitochondrial DNA (Ox-mtDNA) in the cytoplasm binds to GSDMD-NTD at R138, K146, R152, and R154, thereby promoting the oligomerization of GSDMD-NTD in neutrophils and NETs formation 133. Furthermore, activation of the caspase-3-GSDME axis by ROS has been documented in the context of antitumour therapy and chemotherapeutic drug-induced nephrotoxicity 134, 135. Whether ROS modulate pore formation by other GSDMs through oxidative modification requires further investigation.

### Succination

Many studies have revealed that cell metabolism regulates inflammatory responses 136, 137. After being stimulated by LPS, macrophages tend to switch their metabolic characteristics from oxidative phosphorylation to aerobic glycolysis 138, 139. Some metabolic intermediates, such as itaconate, succinate and fumarate, correct aberrant inflammatory responses through transcriptional and metabolic regulation, as well as PTMs 138-140. Moreover, studies have shown that metabolic intermediates regulate various types of cell death, including pyroptosis 138, 141-143. Humphries et al. showed that dimethyl fumarate (DMF) or endogenous fumarate could bind to pivotal cysteine residues on GSDMD and GSDME, resulting in an irreversible process called succination, which forms S-(2-succinyl)-cysteine 138. There are many succination sites on GSDMD and GSDME according to mass spectrometry results. To date, researchers have identified many succination sites in hGSDMD (C56, C191, C268, C309, and C467), mGSDMD (C39, C57, C77, C122, C192, C265, C299, C434, C448, C487, and C489), and human GSDME (C45, C156, C168, C180, C235, C371, C408, C417, and C489) (Table 2). Similarly, succination limits GSDME-induced cell death 138. However, the specific succination sites on GSDME and their impact on its interaction with upstream proteases require further investigation.

### Itaconation

Itaconate contains an electrophilic α, β-unsaturated carboxylic acid moiety that can undergo Michael addition with the cysteine residues of proteins, leading to itaconation 144. Itaconate-associated PTMs have been reported to play pivotal roles in modulating many pathological processes 137. In 2021, Bambouskova et al. showed that endogenous itaconate covalently bound to GSDMD at C77, which was termed itaconation, thereby reducing caspase-1-mediated GSDMD cleavage and establishing tolerance to prolonged periods of LPS exposure in macrophages 141. Significantly, octyl-itaconate or similar itaconate compounds exhibit many PTM sites for the same substrate. Qin and colleagues used the specific and cell-permeable bio-orthogonal probe ITalk to identify additional potential itaconate modification sites for GSDMD (Table 2). Intriguingly, C77 and C192 of GSDMD were shown to be susceptible to itaconate modification. Previous studies have identified C191 in hGSDMD (hC191) and C192 in mGSDMD (mC192) as crucial sites for GSDMD-NT oligomerization 26. These derivatives of itaconate may have a wider range of potential applications than endogenous itaconate in the regulation of pyroptosis. Similarly, further research is needed to determine whether itaconate can modify other GSDMs.

### Palmitoylation

Protein palmitoylation refers to the reversible attachment of palmitic acid to cysteine residues, which participates in multiple intracellular physiological processes. During this process, palmitoyl acyl transferase (PAT) enzymes (zinc finger and DHHC motif-containing) in the ZDHHC family play an indispensable role in transferring palmitate to cysteines 145-147. Accumulating evidence indicates that the palmitoylation of GSDMs is a key regulatory mechanism (Figures 3 and 4). During chemotherapy, pyroptosis induced by GSDME can be enhanced by palmitoylation of the C407 and C408 residues of GSDME-CTD via ZDHHC2, 7, 11 and 15. Palmitoylation inhibitors and palmitoylation site mutation reinforce autoinhibition between GSDME-CTD and GSDME-NTD 147. Recent studies have shown that ZDHHC5/9 mediates ROS-dependent palmitoylation of GSDMD at hC191/mC192 to promote plasma membrane localization, which is indispensable for pyroptosis 145, 146. In addition to GSDMD, GSDMB-FL, GSDME-FL and GSDME-NTD can also be palmitoylated. This means that palmitoylation may serve as a ubiquitous PTM, governing the membrane localization of GSDMs-NTD and thereby regulating pyroptosis. However, the interacting enzymes and the particular modification sites require further study.

### Cysteine modification

hC191/mC192 is essential for GSDMD to mediate pyroptosis, which may also be a target for modulation by other small molecules 26. Disulfiram, an FDA-approved medication for alcohol abuse treatment, can covalently bind to hC191/mC192 within GSDMD through cysteine modification, thereby reducing pore formation in an animal model of LPS-induced sepsis 36 (Figure 3). The Cys-modifying drug necrosulfonamide (NSA) was reported to block pyroptosis by directly binding to hC191/mC192 in GSDMD 37. Collectively, these findings indicate that the modification of key cysteine sites in GSDMs may serve as a pivotal regulatory mechanism of pore formation. This finding highlights the potential therapeutic opportunities of targeting this pathway in various human diseases.

Among these modification residues of GSDMD, hC191/mC192 has been identified as the indispensable target for various PTMs, including oxidation, succination, itaconation and palmitoylation 132, 138, 144-146. Furthermore, several Cys-modifying drugs have shown therapeutic effects in animal models 36, 37. In fact, different PTMs at hC191/mC192 can occur in a context-dependent manner. For example, DMF had little effect on preventing liposome leakage 36. However, DMF inhibited THP-1 cell and BMDM death. In mice, the administration of DMF protected against LPS-induced shock and other inflammatory diseases by targeting GSDMD 138. In addition, there may be interactions between different PTMs during the dynamic process of pyroptosis 145, 146. The palmitoylation of GSDMD-FL and -NTD at hC191/mC192 occurred in a ROS-dependent manner and promoted membrane localization 146. Although multiple PTMs can occur at GSDMD hC191/mC192, the final effect on pyroptosis and the dominant PTMs seems to be associated with the treatment of cells or animals. The competition between different PTMs at GSDMD hC191/mC192 may be the underlying mechanism of pyroptosis regulation.

### Therapeutic strategies involving PTMs

Due to their potential in antimicrobial defence, inflammatory diseases, and cancer treatment, GSDM-mediated pyroptosis has gained significant attention. NSA and disulfiram have shown promising potential in several mouse models, including DSS-induced colitis, LPS-induced sepsis, and vascular remodelling induced by chronic hypoxia 36, 148-151. Itaconate, fumarate and their derivatives exert potential therapeutic effects against inflammatory and pyroptosis-related diseases, as demonstrated by cell and animal models 137, 152. Moreover, several fumarate analogues, including DMF, diroximel fumarate, and tepilamide fumarate, have been FDA-approved for the treatment of multiple sclerosis and autoimmune encephalitis 138. Further exploration is warranted to determine whether these PTM drugs targeting GSDMD could be applied to other diseases caused by pyroptosis. In antitumour therapy, GSDME protein levels are crucial for caspase-3/GSDME-mediated tumour cell pyroptosis. In cases of low GSDME expression, chemotherapy tends to induce apoptosis rather than pyroptosis, thereby attenuating its therapeutic efficacy 119. Furthermore, tumour patients with elevated levels of the pyroptotic GSDMB isoform exhibit improved survival outcomes 68, 69. Activating and stabilizing pyroptotic GSDMs is a valuable strategy for cancer treatment due to their role in antitumour immunity. Achieving the proper balance between cell death and survival is important for antimicrobial defence. To avoid being eliminated, some pathogens release specific effectors that inhibit pyroptosis via PTMs. Therefore, enhancing the activity of GSDMs or eliminating negative effects of pathogens on pyroptosis may exert significant protective effects during bacterial infection.

Overall, applying approved drugs or designing new drugs to target the PTMs of GSDMs to affect signalling shows promising prospects in cancer, infectious diseases and inflammatory diseases.

## Conclusions and future perspectives

In the past two decades, many mysteries of GSDMs have been gradually unveiled. Current studies have demonstrated the underlying mechanism of GSDMs in many diseases. Increasing the activity of pyroptosis aggravates tissue damage and induces an exaggerated inflammatory response, but weakening pyroptosis typically fails to eliminate invading pathogens. Pyroptosis activity is closely related to the sites of cleavage, the types of proteases, and PTMs, which will become optional targets for the future regulation of pyroptosis.

This review focused on describing the latest studies on protease-mediated GSDM cleavage, summarized the underlying sites and described the mechanisms of PTMs. Currently, several questions exist about the cleavage and PTMs of GSDMs. First, as a vital regulatory method, PTMs are involved in most processes associated with GSDM function, including autoinhibition, proteolytic cleavage, oligomerization and even pyroptosis-independent biological effects. Palmitoylation of GSDMD at mC192 occurred in a ROS-dependent manner to regulate membrane translocation, indicating an interplay between different PTMs. Which PTM predominates in specific intermediate steps of pyroptosis and how these PTMs interact remain unclear. Second, it is important to identify what types of GSDMs are expressed in different cells and the changes in GSDMs between normal and diseased states. For GSDMB, it is necessary to determine the main subtype in different cells and tissues. Third, whether we can apply what we know about the PTMs of one GSDM to other GSDMs is worth further examination.

Although the PTMs of GSDMs have been gradually discovered in various diseases, the potential modification pathways and mechanisms are still unclear. Moreover, the PTMs of GSDMs may occur in a context-dependent manner, which indicates that the same PTM may not have the same effects *in vivo* or *in vitro*. For clinical translation, more experiments on the PTMs of GSDMs are needed to determine their effects. Generally speaking, the PTMs of GSDMs are newly emerging targets for modulating cell death and the resulting immune responses.

## Figures and Tables

**Table 1:** Summary of Human and mouse GSDMs functions

GSDMs (Mouse ortholog)		Protease (Cleavage site)	Disease models	Biological function	Reference
GSDMA (Gsdma1, Gsdma2, Gsdma3)		**SpeB (GSDMA: Q246**; **Gsdma1:Q246**; Gsdma2 and Gsdma3: Unknown**)**	Systemic sclerosis, Alopecia, Group A Streptococcus infection	Pyroptosis, mitochondrial damage	21, 22, 38, 57, 60
GSDMB (None)	Isoform1(403aa): Lack exon 6	GzmA (K231) Caspase-1/3/4/6/7/8/9 (D91) NE (M220)	Asthma, Sepsis, Inflammatory bowel disease, Colorectal, Breast, cervical cancers, Shigella flexneri infection	Peri-bronchial smooth muscle and collagen deposition, rs11078928 variant links to deceased asthma risk, Negative regulation the pore-forming function of pyroptotic isoforms	20, 25, 38, 60, 63-73
	Isoform2(394aa): Lack exon 6,7	GzmA (K222) Caspase-1/3/4/6/7/8/9 (D91) NE (M220)		Negative regulation the pore-forming function of pyroptotic isoforms	
	Isoform3(416aa): Normal	**GzmA (**K229**, K244)** Caspase-1/3/4/6/7/8/9 (D91) NE (M220) **Caspase-1(D236)** **Der p3(K244)**		Pyroptosis, Antitumor immunity, Adjuvant GSDMD-mediated pyroptosis (FL)	
	Isoform4(407aa): Lack exon 7	**GzmA (**K229**, K235)** Caspase-1/3/4/6/7/8/9(D91) NE (M220 exon 5)		Pyroptosis, Antitumor immunity	
	IsoformU(411aa): Non-canonical splice junction	GzmA (K233, K239) Caspase-1/3/4/6/7/8/9(D91) NE (M220)		Cell proliferation, Migration, Adhesion (FL), Bacteria killing	
GSDMC (Gsdmc1, Gsdmc2, Gsdmc3, Gsdmc4)		**Caspase-8** **(GSDMC: D240, D365**; Gsdmc1, Gsdmc2 and Gsdmc3: Unknown; **Gsdmc4: D233)** Caspase-6(Unknown)	Breast cancer, Helminth infection	Pyroptosis, Antitumor Immunity, Type 2 immune response, Cytokines secretion	17, 38, 60, 74-76
GSDMD(Gsdmd)		**Caspase-1,4,5,11(GSDMD: D275**; **Gsdmd: D276)** **Caspase-8 (GSDMD: D275**; **Gsdmd: D276)** **NE (GSDMD: C268; Gsdmd: V251)** **CG (Gsdmd: L274)** Caspase-3,7 (GSDMD: D87; Gsdmd: D88) 3C protease (GSDMD: Q193) Nsp5 (GSDMD: Q193)	Sepsis, Inflammatory bowel disease, Bacterial/viral/fungal infection, Kidney diseases, myocardial Ischemia/reperfusion, Non-alcoholic fatty liver disease, Abdominal aortic aneurysm, Rheumatoid Arthritis, Alzheimer's disease, Cancer	Pyroptosis, NETosis, Mitochondrial damage, Bacteria killing, Sublytic release Of inflammatory mediators, Regulate mucus granule exocytosis	15, 16, 31, 38, 41, 60, 77-85, 87-95
GSDME(Gsdme)		**Caspase-3 (GSDME: D270**; **Gsdme: D270)** **GzmB (GSDME: D270**;**Gsdme: D270)**	Autosomal dominant nonsyndromic hearing loss, Neurologic diseases, Atherosclerosis, Aasopharyngeal carcinoma	Pyroptosis, Antitumor Immunity, Sublytic release of inflammatory mediators, Mitochondrial damage	18, 19, 38, 57, 58, 60, 97-104, 153
PJVK(Pjvk)		Unknown	Recessive nonsyndromic hearing impairment	Unknown	1, 38, 48, 60

Table 1: Proteases cleaving and activating GSDMs are marked in bold. The sites in parentheses are proteases cleavage sites. Abbreviations: GzmA: granzyme A; GzmB: granzyme B; NE: neutrophil elastase; CG: cathepsin G

**Table 2:** PTMs regulation of GSDMs

GSDMs	PTM	Factors	PTM sites	PTM effects	Reference
GSDMA	Phosphorylation	PLK1	T8(H)	Inhibit NTD oligomerization	109, 110
	Palmitoylation	Unknown	Unknown	Unknown	146
GSDMB	Ubiquitination	IpaH7.8	K166, K308(H)	Proteasomal degradation	65, 67, 126
			K177, K190, K192(H)	Inhibit membrane translocation (membrane insertion)	
	Dephosphorylation	USP24	Unknown	Unknown	120
	Palmitoylation	Unknown	Unknown	Unknown	146
GSDMC	Phosphorylation	Unknown	S197(H)	Unknown (303 human Ser/Thr kinases potential interaction sites)	154
GSDMD	Phosphorylation	Unknown	S30, S31, T32 (H)	Triple mutation suppresses pryroptosis	112, 154
			S45, S46, S47 (H)	Triple mutation suppresses pryroptosis	
			T213(H)	Enhance steric hindrance to inhibit NTD oligomerization and pyroptosis	
			S181, S185, S201, S250, S252, T251(H)	Unknown (303 human Ser/Thr kinases potential interaction sites)	
	Dephosphorylation	PP1	T213(H)	Promote NTD oligomerization	112
	Ubiquitination	IpaH7.8	K55, K62, K204(H)	Proteasomal degradation	33, 126
	K27-Polyubiquitination	SYVN1	K203, K204(H)	Promote pyroptosis	114
	Oxidation	ROS	C38, C56, C268, C467(H) C39, C57, C265, C487(M)	Promote cleavage by caspase-1	131, 132
			C192(M)	Promote NTD oligomerization	
	Itaconation	Endogenous itaconate	C77(M)	Reduce proteolytic cleavage	141, 144
		ITalk	C77, C192(M)	Unknown (Similar to Itaconate)	
	Succination	Fumarate derivatives (MMF, DMF)	C191(H)/C192(M)	Reduce proteolytic cleavage and inhibit NTD oligomerization	138
	Palmitoylation	ZDHHC5/9	C191(H)/C192(M)	Promote plasma membrane localization	145, 146
	Cysteine-modification	Disulfiram	C191(H)/C192(M)	Inhibit NTD oligomerization	36
	Cysteine-modification	Necrosulfonamide	C191(H)/C192(M)	Inhibit NTD oligomerization	37
GSDME	Phosphorylation	AMPK	T6(H)	Inhibit cleavage by caspase-3 and NTD oligomerization	109, 111, 154
		Unknown	S252, T117, T6(H)	Unknown (303 human Ser/Thr kinases potential interaction sites)	
	Deubiquitination	OTUD4	Unknown	Maintain the stability of GSDME	117
	Stabilization (Deubiquitination)	ORFV	Unknown	Maintain the stability of GSDME	119
	K48-link deubiquitination	USP48	K120, K189(H)	Maintain the stability of GSDME	118
	Succination	Fumarate derivatives (MMF, DMF)	C45(H)	Inhibit Pyroptosis (Similar to GSDMD)	138
	Palmitoylation	ZDHHC2/7/11/15	C407/C408(H)	Promote pyroptosis (CTD is palmitoylation)	147
	Palmitoylation	Unknown	Unknown	Most likely promote plasma membrane localization (NTD is palmitoylation)	146

Table 2: The PTMs sites of human or murine proteins are indicated in H or M in parentheses. Abbreviations: Polo-like kinase 1: PLK1; PP1: Phosphatase1; SYVN1: Synoviolin; ROS: reactive oxygen species; ITalk: a specific and cell permeable bioorthogonal probe; MMF: Monomethyl fumarate; DMF: Dimethyl fumarate; OTUD4: ovarian tumor family deubiquitinase 4; ORFV: oncolytic parapoxvirus ovis

## Abbreviations

GSDMs: gasdermins
PTMs: posttranslational modifications
NTD: N-terminal domains
CTD: C-terminal domains
MLKL: mixed lineage kinase domain-like
SpeB: Streptococcus pyogenes exotoxin B
GzmA: granzyme A
GzmB: granzyme B
NE: neutrophil elastase
CG: cathepsin G
SYVN1: Synoviolin
NSA: necrosulfonamide
PP1: Phosphatase1
ROS: reactive oxygen species
ZDHHC: zinc finger and DHHC motif-containing family
PKA: Protein kinase A
AMPK: AMP-activated protein kinase
Ox-mtDNA: Oxidized mitochondrial DNA
PLK1: Polo-like kinase 1
HDMs: house dust mites
NE: neutrophil elastase
CG: cathepsin G
α-KG: α-ketoglutarate
DR6: death receptor 6
EV71: Enterovirus 71
OTUD4: ovarian tumor family deubiquitinase 4
ORFV: oncolytic parapoxvirus ovis
IBD: inflammatory bowel disease
